# Cement pavement void detection algorithm based on GPR signal and continuous wavelet transform method

**DOI:** 10.1038/s41598-023-46752-2

**Published:** 2023-11-12

**Authors:** Qiuqin Yu, Youxin Li, Tingyi Luo, Jun Zhang, Liang Tao, Xin Zhu, Yun Zhang, Liufen Luo, Xinxin Xu

**Affiliations:** 1 Guangxi Beitou Highway Construction Investment Group Co., Ltd., Guangxi, 5300281 China; 2 National Engineering Laboratory for Highway Maintenance Equipment, Chang’an University, Xi’an, 710064 China; 3 Key Laboratory of Road Construction Technology and Equipment of Ministry of Education, Chang’an University, Xi’an, 710064 China; 4 AVIC Xi’an Aircraft Industry Group Company Ltd, Xian, 710089, China; 5 Guangxi Transportation Science and Technology Group, Nanning, 530007 China

**Keywords:** Geomagnetism, Geophysics

## Abstract

The dimension of the void area in pavement is crucial to its structural safety. However, there is no effective method to measure its geometric parameters. To address this issue, a void size extraction algorithm based on the continuous wavelet transform (CWT) method was proposed using ground-penetrating radar (GPR) signal. Firstly, the finite-difference time-domain (FDTD) method was used to investigate the GPR response of void areas with different shapes, sizes, and depths. Next, the GPR signal was processed using the CWT method, and a 3D image based on the CWT result was used to visualize the void area. Based on the differences between the void and normal pavement in the time and frequency domains, the signal with maximum energy from the CWT time–frequency result was extracted and combined to reconstruct the new B-scan image, where void areas have energy concentration phenomenon. Based on this, width and depth of void detection algorithm was proposed to recognize the void area. Finally, the detection algorithm was verified both in numerical model and physical lab model. The results indicated that the CWT time–frequency energy spectrum can be used to enhance the void feature, and the 3D CWT image can clearly visualize the void area with a highlighted energy area. After fully testing and validating in numerical and lab models, our proposed method achieved high accuracy in void width and depth detection, providing a precise method for estimating void dimension in pavement. This method can guide DOT departments to carry out pre-maintenance, thereby ensuring pavement safety.

## Introduction

The cement pavement subjects to void problem, namely gap between slab and base, due to uneven construction, uneven settlement of subgrade, and the influence of vehicle load and temperature stress. The geometric size of the void area is vital for the bearing capacity of slab. With the size increasement of the void area, the void leads to the settlement and misalignment of the cement slab, and eventually causes the slab breaks^1^. Therefore, it is urgent to develop a non-destructive testing (NDT) method to determine the void area and give an accurate maintenance guide before the slab broken.

Currently, a wide range of methods have been developed for void detection, including impulse response technology^2,3^, acoustic vibration method^4^, distributed optical vibration sensing method^5^ and ground penetrating radar (GPR) method^6,7^. Among them, GPR is the most effective NDT technology in pavement void detection, and GPR has been widely used in detecting pavement defect^8^, structural thickness^9^, rebar^10,11^, and etc. Through numerical analysis, Li et al. found that the width of the void area affects the bearing capacity of the structure, while the void thickness doesn’t affect^12^. To obtain the width of the void area, the newly developed deep transfer learning method provide a quick method to locate the void area in the GPR image. For example, our work adopted the hybrid ResNet50-YOLO model to identify the width of moisture damage in asphalt pavements^13^, and the shallow ResNet18-YOLOv2 model with IRS methods were adopted to locate the void range in airport runway^11^. Besides, Yan et al. used Faster-CNN network to detect pavement crack, and combined the morphological method to calculate the length and width of crack area^14^. The image-based deep learning method provides a new method for the interpretation of GPR data; however, it requires a large number of B-scan images for network training, and subjects to the problem of determining the boundary of target area.

A-scan, which is the basic signal unit in GPR system, contains target information such as depth, layer thickness and material dielectric property. Thus, the GPR signal can be used for identifying boundary of defect area. For example, we surveyed the bridge deck asphalt pavement with a high-frequency 2.3 GHz antenna, and 28 time and frequency domain statistical parameters from A-scan signal were extracted to train an ANN model to locate moisture damage area^15^. Similarly, 28 time and frequency domain parameters and PCA method were also adopted to classify void and normal pavement in our another work^16^. The joint time–frequency parameters provide a new view for identification of underground target, but the selection of sensitive feature parameters is affected by human experience. In contrast, the time–frequency domain transform method can obtain richer information than previous statistical parameters. Dai et al. used short-time Fourier transform (STFT) to improve the interpretation accuracy of GPR signals with a Hamming window based on an integer multiple of the radar wavelet length^17^. STFT subjects to the problem of determining the window width, which affects the signal resolution.

To overcome the shortcoming of STFT, Wu et al. used continuous wavelet transform (CWT) to analyse the GPR signals in the airport runway, and found that the void size could be determined according to the CWT energy^18^. He et al. used the S-transform spectral inversion optimization algorithm to process the GPR data of the airport roadway, and effectively estimated the thickness of the thin void layer ^19^. In addition, Liu et al. adopted deconvolution method to extract thickness dimension, and proposed a sparse pulse deconvolution algorithm to extract reflection coefficient sequences, which effectively improved the longitudinal resolution of GPR data^20^. Zhao et al. used the L-curve method to determine the parameters of regularized deconvolution, and predicted the thickness of thin asphalt^21^. Jazayeri et al. proposed a sparse blind deconvolution algorithm to improve the precision extraction of geometric parameters in target regions^22,23^. Existing studies have shown that both the time–frequency statistical parameters and time–frequency transform of GPR signal can be used to estimate the thickness of the underground target area, but there is no research on using the joint time–frequency parameters to determine the geometrical parameters of the void area.

To address the above issue, a void width and depth extraction method was proposed by using CWT method. FDTD method was adopted to simulate the void area in different situations to get the ground truth of void and normal pavement signal. CWT was used to process the GPR signal, based on the energy difference between void and normal pavement in CWT results, a reconstruction energy spectrum method was proposed to estimate the width and depth of void areas. The void geometric parameter detection algorithm was tested and verified both in numerical and lab model. The research results, including void width and depth parameters, can be used to evaluate the bearing capacity and residual life of pavement structures, which is crucial for making premaintenance plan of pavement.

## GPR signal processing principle

### F-K migration algorithm

According to the GPR imaging principle on a single object, hyperbolic curve will be formed on both sides of the target. However, the envelope lines are not the position of the object. The F-K migration algorithm can effectively focus the scattered energy and can eliminate and suppress the hyperbolic effect. Its principle is based on the fact that the electromagnetic (EM) wave emitted by the GPR will undergo multiple refractions and reflections underground. Each reflection point is used as a sub wave source that emits EM waves from zero time. The migration results can be obtained by inversion of the echo data. It uses the reflection source model to solve the wave Eq. (1), in which the reflection signal field is generated by the reflection at the position of the object. F-K migration method calculates the wave field when reflection occurs and the wave is still at the reflection source t = 0. The essence of F-K migration is Fourier transform, which is derived from the general summation expression of wave function as Eq. (2)^24,25^.

1 $$\left( {\frac{{\partial^{{2}} }}{{\partial {\text{x}}^{{2}} }}{ + }\frac{{\partial^{{2}} }}{{\partial z^{{2}} }} + \frac{1}{{v^{2} }}\frac{{\partial^{{2}} }}{{\partial t^{{2}} }}} \right)\varphi \left( {x,z,t} \right) = 0$$

2 $$\varphi \left( {x,z,t} \right) = \frac{1}{2\pi }\int_{ - \infty }^{ + \infty } {\int_{ - \infty }^{ + \infty } {\varphi \left( {k_{x} ,\omega } \right)e^{{ - j\left( {k_{x} x + k_{z} z - \omega t} \right)}} dk_{x} d\omega } }$$

where $v$ is the propagation velocity of EM waves, $k_{x}$ and $k_{z}$ are wave-numbers in the *x* and *z* directions; $\omega = \left( {v/2} \right)\sqrt {k_{x}^{2} + k_{y}^{2} }$ represents the frequency; and $\varphi$ is the Fourier transform from the surface field $\varphi \left( {x,0,t} \right)$. $\varphi \left( {x,0,t} \right)$ equals to the measured signal $\Upsilon \left( {x,t} \right)$ when GPR acquires the waves propagated to the pavement surface. The target is estimated by the initial wave-field $\text{I}\left( {x,z} \right)$ at $t = 0$ as following.

3 $$\text{I}\left( {x,z} \right) = \varphi \left( {x,z,0} \right) = \frac{1}{2\pi }\int_{ - \infty }^{ + \infty } {\int_{ - \infty }^{ + \infty } {\varphi \left( {k_{x} ,\omega } \right)e^{{ - j\left( {k_{x} x + k_{z} z} \right)}} dk_{x} d\omega } }$$

By resampling $\varphi \left( {k_{x} ,\omega } \right)$ to the $k_{x} - k_{z}$ domain, the migration results can be calculated by the inverse fast Fourier transform $F^{ - 1}$. In our work, F-K migration and the following GPR signal process are all processed by home-made MATLAB program, and the parameter of propagation velocity can be calculated as following.

4 $$\text{I}\left( {x,z} \right) = F_{{k_{x} }}^{ - 1} F_{{k_{z} }}^{ - 1} \phi \left( {k_{x} ,k_{z} } \right)$$

5 $$\phi \left( {k_{x} ,k_{z} } \right) = \frac{{vk_{z} }}{{\sqrt {k_{x}^{2} + k_{z}^{2} } }}\phi \left( {k_{x} ,v\sqrt {k_{x}^{2} + k_{z}^{2} } } \right)$$

F-K migration is a linear function since the Fourier transform satisfies the linear super-position principle. The migration results of radargram can be regarded as the summation of migration from all Stationary wavelet transform (SWT) coefficients as following.

6 $$M\left( {\Upsilon \left( {x,t} \right)} \right) = \sum\limits_{i = 1}^{N} {\left( {M\left( {DH_{i} } \right) + M\left( {DV_{i} } \right) + M\left( {DD_{i} } \right) + M\left( {A_{N} } \right)} \right)}$$

where $M\left(\cdot \right)$ represents the F-K migration function. The SWT coefficients have a good time–frequency resolution, and each component can contain clutter, targets, or hyperbola interference. Effective SWT signals containing the targets can enhance the migration results, while those occupied by clutter or hyperbola interference should be discarded. Thus, the final target profile is reconstructed by selected migration components.

### Continuous wavelet transform

GPR signal belongs to transient non-stationary signal. The traditional Fourier transform cannot reveal the distribution of different frequency components in the time domain due to the limit characteristics of its basis function. CWT is a time–frequency localization analysis method with fixed window size and changeable shape, which captures the local and global characteristics of signals through different scales. CWT is often used for time–frequency analysis or transient signal location, especially for signals with sudden change of instantaneous frequency^24^, such as electrocardiogram (ECG) signal diagnose analysis. Continuous wavelet coefficient matrix is extracted from time domain to time–frequency energy spectrum analysis, which is adopted to enhance the characteristics of void area. According to our previous analysis, the GPR feature of the void and normal area in time domain and frequency domain are quite different^16^, usually, the amplitude in void area would be larger than that in normal pavement both in time and frequency domain. Therefore, when the GPR signal is converted from time domain to time–frequency domain, the signal from void area would present high energy than that of normal pavement, this could help us locate the void area.

The expression of CWT is as following.

7 $$WT_{{_{x} }} \left( {b,a} \right) = < x\left( t \right),\psi_{{_{a,b} }} \left( t \right) > = \frac{1}{\sqrt a }\int\limits_{ - \infty }^{ + \infty } {x\left( t \right)\psi^{*} \left( {\frac{t - b}{a}} \right)dt}$$

where $x\left( t \right)$ is the GPR signal and $\psi_{a,b} \left( t \right)$ is the mother wavelet. *a* and *b* are the scale and translation parameters of the mother wavelet, respectively. Therefore, wavelet transform is a multi-resolution analysis method that projects the signal onto a series of wavelet basis functions generated by the mother wavelet.

According to the inner product theorem of wavelet transform, the weighted sum of the amplitude squared of wavelet transform in time and frequency domain is equal to the total energy of the signal in time domain, which was described in Eq. (8). Therefore, the amplitude square of wavelet transform can be used as another form of time–frequency distribution of signal energy.

8 $$\int_{ - \infty }^{\infty } {\left| {x\left( t \right)} \right|^{2} dt = \frac{1}{{c_{\psi } }}\int\limits_{0}^{\infty } {\int_{ - \infty }^{\infty } {\left| {WT_{x} \left( {b,a} \right)} \right|^{2} } } } dadb/a^{2}$$

9 $$c_{\psi } = \int_{0}^{\infty } {\psi \left( \omega \right)/\omega } \, d\omega$$

where $\psi \left( \omega \right)$ is the Fourier transform of $\psi \left( t \right)$.

Considering a good balance between localization in time and frequency, and extracting more feature information, the mother wavelet adopts nonorthogonal complex Morlet (or amor wavelet named in MATLAB) wavelet, which is a Gaussian function with zero mean and modulated by complex cosine. The waveform of mother wavelet has the characteristics of energy attenuation and local convex peaks, which can achieve a good matching to the GPR Ricker reflection wavelet. The time domain analytic function of the mother wavelet is defined as follows.

10 $$\psi \left( t \right) = e^{{ - t^{2} /2}} \cos 5t$$

## Void geometric parameters extraction algorithm

### Reconstructed energy spectrum

The GPR uses spherical wave to transmit and receive signal, therefore, GPR signal would contain many clutters. To improve the signal-to-noise ratio (SNR) of GPR signal and enhance the characteristics of void signal, dedicated GPR signal processing methods were employed, including static correction, energy gain, background removal, band-pass filtering, and F-K migration method.

According to the GPR propagation principle, if the EM wave meets the reflecting surface with dielectric difference during the propagation process, EM wave will be reflected. The larger the reflection index is, the stronger the energy of the reflected wave is. Ricker wavelet has the characteristics of local energy attenuation. The energy and frequency of the reflected wavelet are closely related to the size of the target area and the difference of the medium.

**Figure 1:**
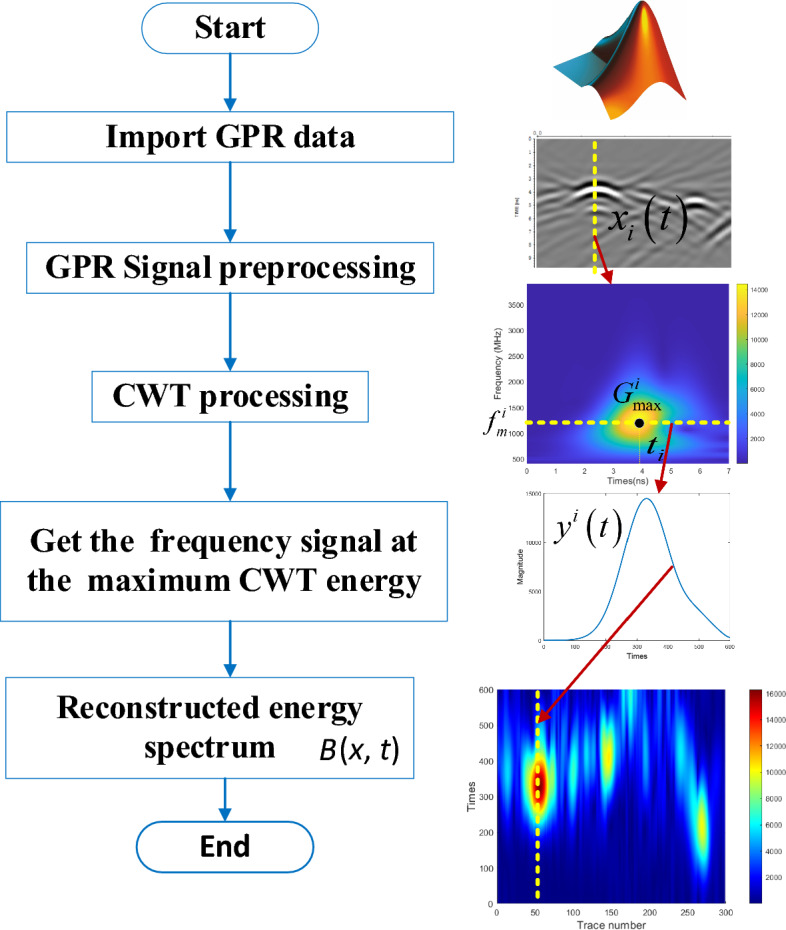
Reconstructed energy spectrum flow chart.

Due to the target area presents large amplitude or high energy phenomena, we proposed a CWT energy reconstructed method by extracting the single frequency signal information corresponding to the maximum energy in the CWT result. The processing process is shown in Fig. 1. Taking the *i*-th A-scan $x_{i} \left( t \right)$ from processed GPR data, CWT method was used to transform it from time domain to time–frequency domain, where its wavelet coefficient spectrum is $G^{i} \left( {f,t} \right)$. If this GPR signal contains a target, we will obtain a maximum value from CWT results denoted as $G_{\max }^{i}$ at the target depth area. The frequency component $f_{m}^{i}$ corresponding to maximum $G_{\max }^{i}$ is taken as the main frequency from CWT result and was extracted as new A-scan $x_{i} \left( t \right)$. Repeating the reconstruction process for the all the traces in GPR data, we got a new reconstructed signal B-scan $B\left( {x,t} \right)$, which is the stacking of all new A-scan with maximum frequency from CWT result. Compared new reconstructed B-scan with the original B-Scan in Fig. 1, one can observe that the reconstructed energy spectrum $B\left( {x,t} \right)$ has higher resolution, which could effectively reveal the horizontal and vertical distribution of void areas area.

### Energy function probability of the void area

**Figure 2:**
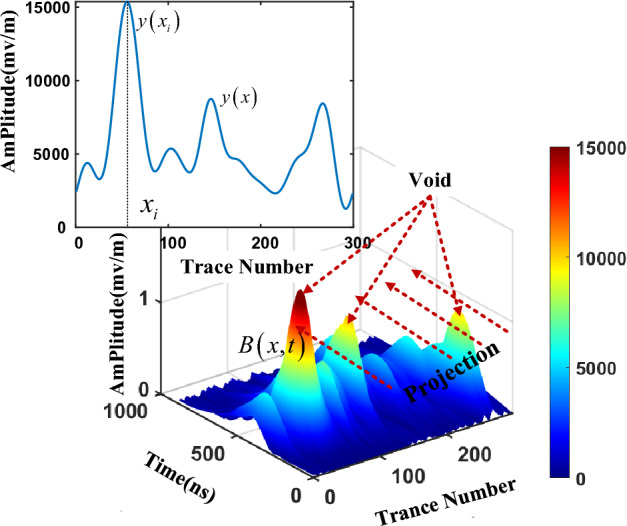
Reconstructed energy spectrum projection curve.

3D visualization of the energy spectrum $B\left( {x,t} \right)$ can effectively highlight target area and reveal the spatial energy distribution of the entire data. The 3D visualization of reconstruction B-scan was shown in Fig. 2, where void areas present local convex peak features. The convex peak is the energy concentration area, which can represent the location and width of the void area. To obtain the void dimension from reconstruction signal more easily, we reduce the dimension of the 2D matrix $B\left( {x,t} \right)$ into 1D energy function $y\left( x \right)$. Thus, the local convex peak of the void becomes the local extreme point of the curve *y*, and the maximum points can be further used to determine the position of the void. The dimensionality reduction process could be expressed as

11 $$y\left( {x_{i} } \right) = B_{\max } \left( {x_{i} ,t} \right)$$

where $y\left( {x_{i} } \right)$ is the maximum value of energy spectrum at *i*-th trace $x_{i}$, and *N* is the A-scan number in B-Scan. $y\left( {x_{i} } \right)$ is the maximum value of *i*-th trace that makes up the maximum vector $y\left( x \right)$, which is described as following.

12 $$y\left( x \right) = \left[ {y\left( {x_{1} } \right),y\left( {x_{2} } \right), \cdots ,y\left( {x_{N} } \right)} \right]$$

**Figure 3:**
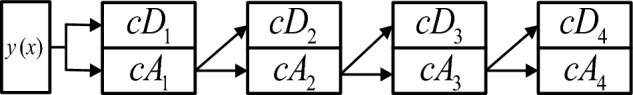
Schematic diagram of wavelet decomposition.

The smoothness of $y\left( x \right)$ is affected due to noise signal. To smooth the curve, the Symlets 4 wavelet (sym4) is used for the signal $y\left( x \right)$ and a 4-layer discrete wavelet transform (DWT) is performed. The decomposition process is shown in Fig. 3. The algebraic relationship between the original signal and the decomposed wavelet coefficients is shown in Eq. (13).

13 $$y\left( x \right) = \sum\limits_{i = 1}^{n} {cD_{i} } + cA_{n}$$

where $cD_{i}$ is the high-frequency wavelet coefficient, $cA_{n}$ is the low-frequency wavelet coefficient. *n* is the number of decomposition layers, the smaller *n* is, the lower the frequency band resolution is, and vice versa.

**Figure 4:**
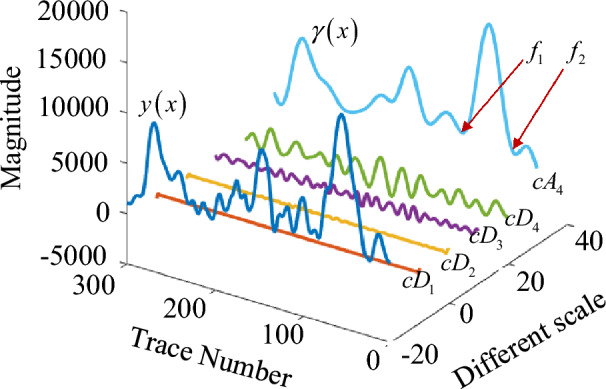
Decomposition signal after 4-layer DWT decomposition.

Figure 4 is the result curve after 4-layer decomposition by DWT method. Compared with the raw signal, the decomposed approximate signal $\gamma \left( x \right)$ (or $cA_{4}$) does not affect the position of the extreme value point of curve $y\left( x \right)$ and can make the curve smoother. Therefore, we could directly get the extreme points from signal $\gamma \left( x \right)$ to determine void area.

In Fig. 4, decomposition signal $\gamma \left( x \right)$ has three void areas. Each void area has convex peak feature and contain one peak area and two edges, i.e., the local minimum points $f_{1}$ and $f_{2}$ are the two edges of void area. If we want to determine the void width, we only need to find the two local minimum points. However, due to the interference of background noise, the local minimum value may not be a real local minimum point of void area, which would cause width error. Therefore, it is necessary to set a threshold to determine whether this point is the local minimum point of void area or not.

Assuming that the energy distribution approximately of sample signal $\gamma \left( x \right)$ obeys the lognormal distribution. The energy probability density function is shown in Eq. (14). Therefore, the expected value $E(\gamma )$ can be used as the threshold, which can be determined from Eq. (15).

14 $$f_{\lg - N} (\gamma ;\mu ,\sigma ) = \frac{1}{{\gamma \sigma \sqrt {2\pi } }}e^{{ - \frac{{(\ln \gamma - \mu )^{2} }}{{2\sigma^{2} }}}}$$

15 $$E(\gamma ) = e^{{\mu + \sigma^{2} /2}}$$

where $\mu$ and $\sigma$ is the mean value and standard deviation of logarithm of $\gamma \left( x \right)$, respectively.

**Figure 5:**
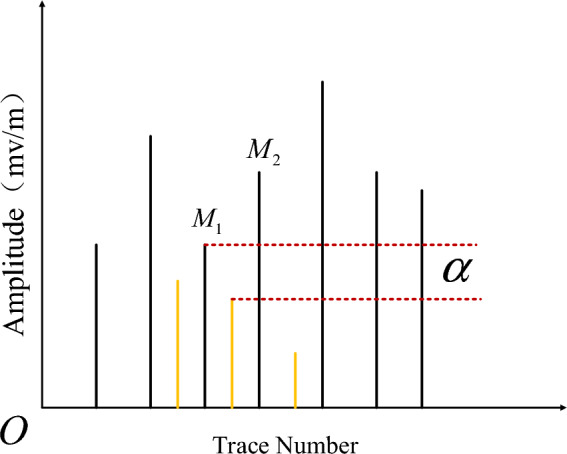
Distribution of extreme points.

Due to GPR signal has hyperbolic interference phenomena, it would cause pseudo void points between the two void areas, these points need to be filtered. In Fig. 5, there have multiple pseudo local points, whose value is $y_{\min }$ with yellow line. We introduced a relative height factor $\alpha$ to determine whether this point is real or pseudo point, and it is defined as following.

16 $$\alpha = \min \left( {M_{1} ,M_{2} } \right) - y_{\min }$$

where $M_{1} ,M_{2}$ are the two extreme points on the left and right of the local minima point of minimum value $y_{\min }$. When $\alpha > 0$, this local point is caused by hyperbolic interference and should be deleted, otherwise, we keep this point as edge of void aera.

### Void width extraction algorithm

**Figure 6:**
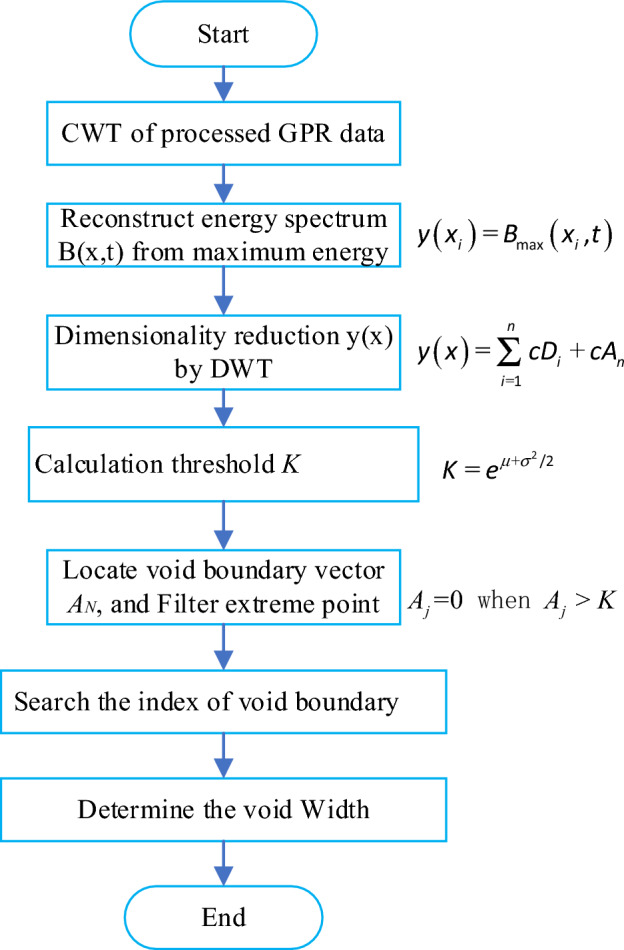
Width extraction flow chart from CWT reconstruction result.

The void width extraction algorithm is shown in Fig. 6, and the extraction step is as following.

1. Obtain 1D dimensionality reduction energy function. B(x,t) is the reconstruct matrix with void area according to the flowchart of Fig. 1, and the energy distribution curve $T\left( t \right)$ is calculated according to B(x,t). The 1D dimensionality reduction energy function $y\left( x \right)$ was obtained according to Eqs. (10) and (11).
2. Find the threshold value. Estimate the lognormal distribution of each sample $\gamma \left( x \right)$ and determine the threshold value $K$ according to Eqs. (14) and (15).
3. Locate the void boundary point. Find all the maximum points of the function $\gamma \left( x \right)$ and store their values to form a vector $A_{N}$, *N* is the trace number. Set the vector value $A_{j}$ = 0 when $A_{j}$ > K.
4. Search the index of void boundary. Filter the interface points between two adjacent void area, search all the local minimum point $\varphi \left( x \right)$ from $A_{N}$ and calculate their mean value as $H_{mean}$,if $\varphi \left( {x_{i} } \right) > H_{mean}$, let $\psi \left( i \right) = 0$. After this, the vector $\psi \left( m \right)$ saves the index of extreme points from $A$, and $m$ is the number of all extreme points.
5. Determine the void width. According to the index of $\psi \left( m \right)$, find the two nearest minimum points on the left and right of the maximum point in the function $y\left( x \right)$ to determine the width boundary.

**Figure 7:**
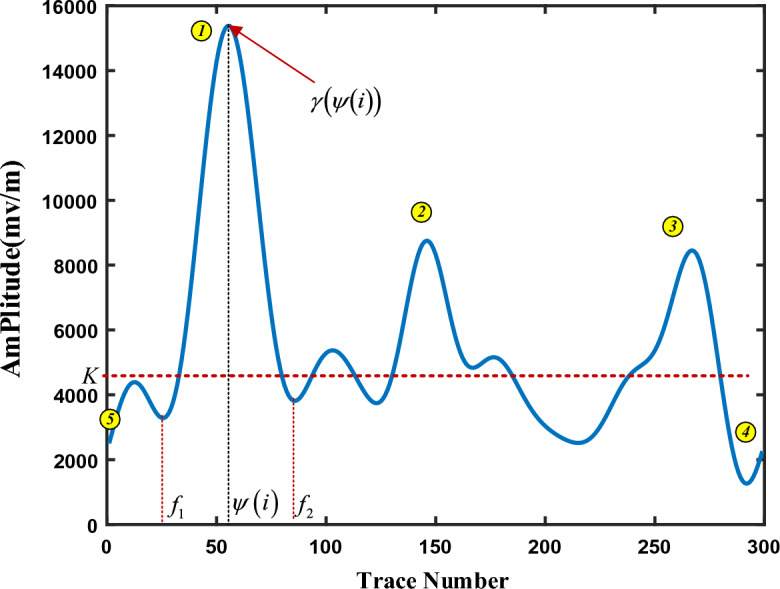
Void width searching algorithm.

CWT method is performed on the processed GPR data according to the process in Fig. 6, DWT with the sym4 wavelet function is used to obtain the curve $\gamma \left( x \right)$, which is shown in Fig. 7. Based on the extraction algorithm, we got the trace index of extreme point array $\psi \left( x \right)$. For example, we find the trace number of maximum points at Point 1, and two nearest local minimum points, denoted as $f_{1} ,f_{2}$. The void width can be obtained from the corresponding track distance between $f_{1}$ and $f_{2}$. To locate the two local minimum point, a threshold limit needs to be set to avoid overlapping. And the boundary points 4 and 5 should be deleted by adding constrained condition. Similarly, the width of void area near point 2 and 3 can also be determined by the same method.

### Void depth extraction algorithm

Due to the edge of void area are not easy to locate due to the resolution of EM wave, here, we take the maximum energy position in depth (or sample point) as the upper depth of void area. Given that we have obtained the void width and stored in vector $L_{m}$, which has been determined by width extraction algorithm. To improve the resolution in time axis, here, we adopted S-transform $Gs^{i} \left( {f,t} \right)$ to process the A-scan data in $L_{m}$^26,27^, search all the maximum point in energy function $g_{i} \left( t \right)$ for each void trace and combine a depth vector g(t).

17 $$g_{i} \left( {t_{n} } \right) = \max \left( {abs(Gs^{i} \left( {f,t_{n} } \right))} \right),n = 1,2, \cdots ,N;i = 1..m$$

18 $$g\left( {t,i} \right) = \left[ {g_{1} \left( {t_{1} } \right), \cdots ,g_{m} \left( {t_{m} } \right)} \right]$$

where $g_{i} \left( {t_{n} } \right)$ is the maximum energy value at time $t_{n}$ in *i*-th trace, $g\left( {t,i} \right)$ is the depth vector for void area.

**Figure 8:**
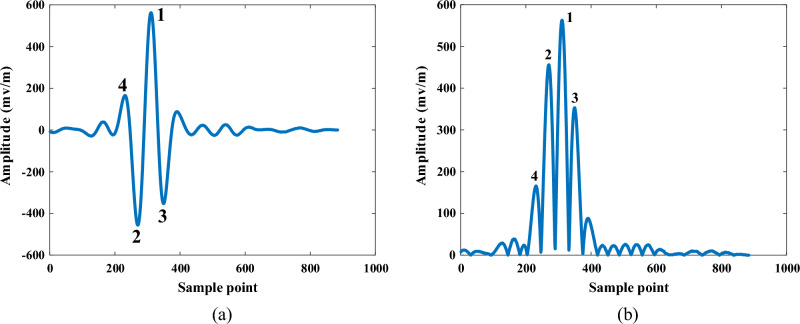
Depth location in S-transform energy dimensionality reduction signal. (**a**) Ricker wave. (**b**) Absolute value of void S-transform signal.

If there is a different dielectric layer underground, there would be a reflected Ricker wavelet, the first sub-wavelet peak of Ricker wavelet should be the upper depth of void area. Take one S-transform void signal for example, Fig. 8a shows the reflection ricker wave at void area, where point 1#, 2# and 3# are the typical peak point. Point 2# was used to refer the boundary of different media in A-scan signal. The upper depth could be determined by the velocity or dielectric constant (19).

19 $$d = {{c \cdot t} \mathord{\left/ {\vphantom {{c \cdot t} {2\sqrt \varepsilon }}} \right. \kern-0pt} {2\sqrt \varepsilon }}$$

where $t = m \cdot dt$, *m* is the sample point index; *dt* is sample interval time of GPR, $\varepsilon$ is the dielectric constant of concrete pavement.

Figure 8b is the absolute value from one void S-transform signal, all the values are converted to positive value. According to the Ricker wave feature, the upper boundary should be the second extreme value, therefore, the void depth could be determined by searching the peak wave points.

## Algorithm validation

### Numerical model

**Figure 9:**
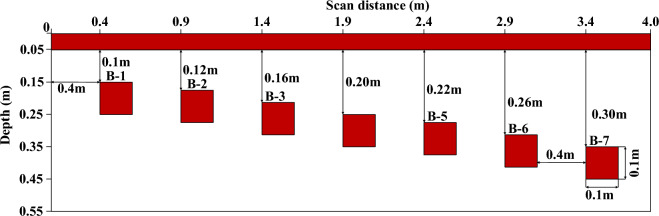
Schematic diagram of the void model.

Cement pavement model with 7 rectangle air void areas were created and shown in Fig. 9, where all the void areas have the same dimension with 0.1 m × 0.1 m, while their depth ranges from 0.1 to 0.3 m. Simulation was processed in gprMax 3.15 and the simulation parameters are shown in Table 1. The central frequency of the GPR antenna is 800 MHz, the wavelength of the EM wave in the air void area is $\lambda = 0.375$ m.

**Table 1:** FDTD forward simulation parameters.

Parameter type	Value
Model size (m^2^)	4.0 × 0.55
Sample interval (m)	0.005
Time window (ns)	12
Antenna spacing (m)	0.14
Antenna frequency (MHz)	800
Excitation source type	Ricker
Trace number	694

### CWT feature of void area

**Figure 10:**
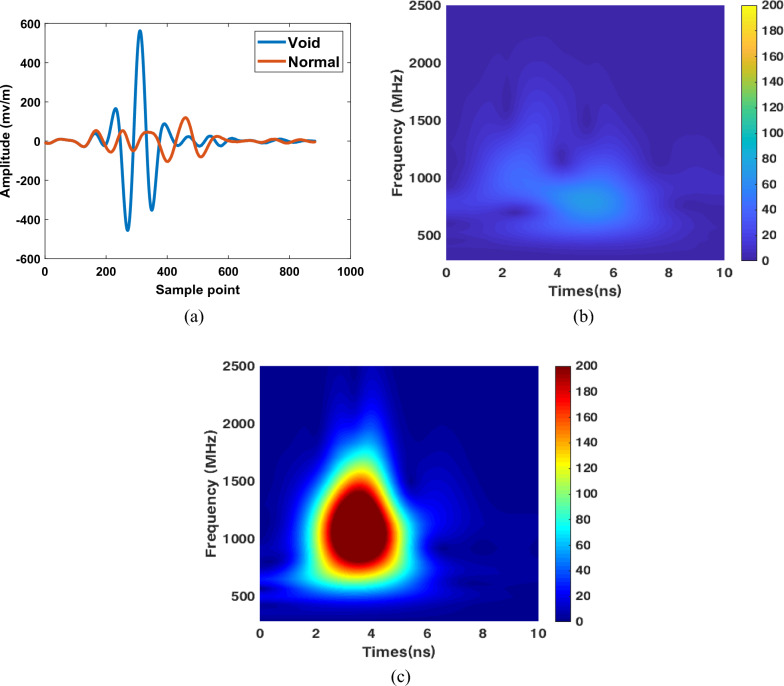
CWT Comparison result between normal and void signal. (**a**) Comparison of normal and void signal in time domain. (**b**) CWT result of normal trace. (**c**) CWT result of void trace.

Due to the numerical result doesn’t have noise, therefore, only F-K migration method was used to process the raw data and reduce the hyperbolic phenomenon. GPR signal of normal and void area from simulated model was used for comparison and shown in Fig. 10. It is obvious that the time frequency spectrum between normal and void area are quite different. The CWT result of normal signal is uniform without energy concentration, while the void CWT result has energy concentration area, the energy concentration area reflects the void position.

**Figure 11:**
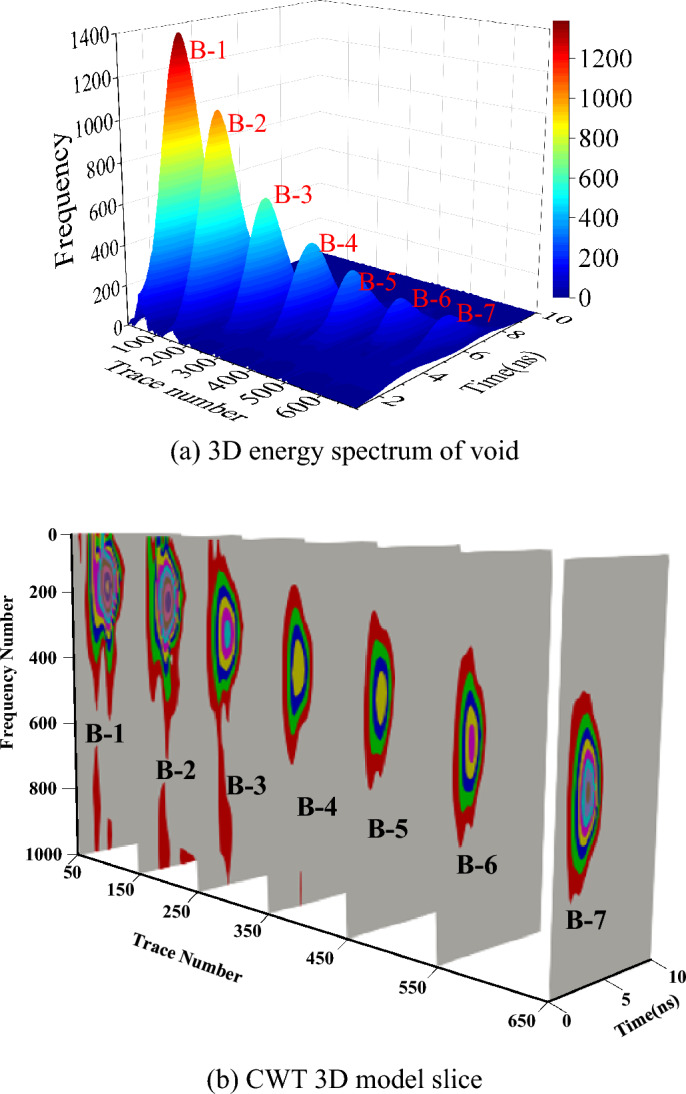
3D visualization of disease area. (**a**) The 3D energy spectrum of model. (**b**)The CWT slice at void area.

All the GPR data from simulated model was processed by CWT and further be combined in 4D CTW matrix. To view the result more easily, Paraview software was used to for 3D view of void area, where the colour represents the amplitude. Therefore, the CWT matrix was exported to VTK file from MATLAB to Paraview. Figure 11a shows the 3D visualization of the time–frequency spectrum of the void area. It can be seen that void areas from B-1 to B-7 are obvious. The deeper the void area, the less energy of CWT result. To view more clearly of void area, traces from 7 central void areas were extracted, and its CWT slice was shown in Fig. 11b, where the 7 void areas could be visualized. This indicates that the CWT can enhance the difference between void and normal pavement, however, it is not easy to determine the boundary of void area just from CWT result.

**Figure 12:**
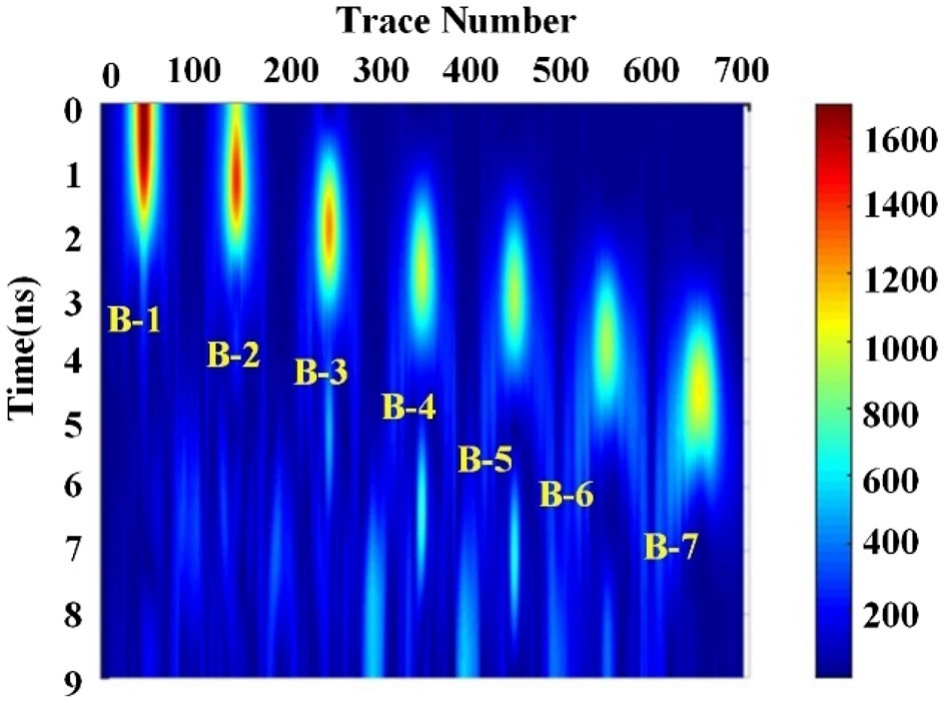
Reconstructed energy spectrum.

To view the void feature in 2D view, we reconstruct CWT result according to the flow chart in Fig. 1. The reconstructed energy spectrum is shown in Fig. 12. Compared with simulated model of Fig. 9 with the reconstructed spectrum of Fig. 12, one can easily observe the void area in CWT spectrum, and the reconstructed spectrum is more suitable to present the void shape than that in Fig. 11.

It can be clearly seen that void areas appear in concentration area in Fig. 12. The reason is that the echo energy of the normal signal is low and there is no reflected Ricker sub-echo, so the CWT values of normal area are small, thus, the normal pavement becomes the background noise, which could be filtered to enhance the void area. While the GPR signal appears strong echo in the void area, which highlights void areas in the time–frequency energy spectrum. In addition, the vertical distribution of the concentrated band of the reconstructed energy spectrum reflects the depth information of void area, while the horizontal distribution reflects the width information of the void area, later, we will test our proposed method for width and height detection.

### Algorithm validation in numerical model

**Figure 13:**
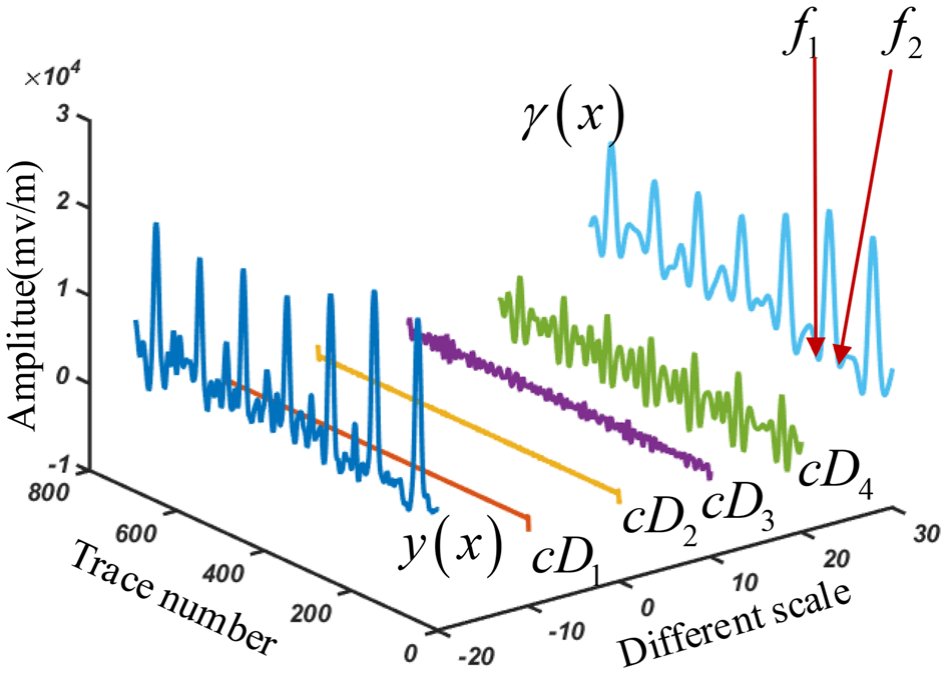
DWT decomposition signal from energy spectrum.

To calculate the void range, 4-layer DWT is used to eliminate the background noise interference for data in Fig. 12. The processed result is shown in Fig. 13. We got the decomposed approximate signal $\gamma \left( x \right)$, which is same to cA4. According to the width extraction algorithm, we firstly search all the extreme points, then filter the interface points by setting threshold value *K*. Finally, we get the boundary index and determine the void width according to $\left( {f_{1} - f_{2} } \right) \times dx$, dx is the sample interval. To prevent the minimum point coincidence between the void area, the energy threshold is set as $K = 7895.2$ according to the $\gamma \left( x \right)$ sample.

**Figure 14:**
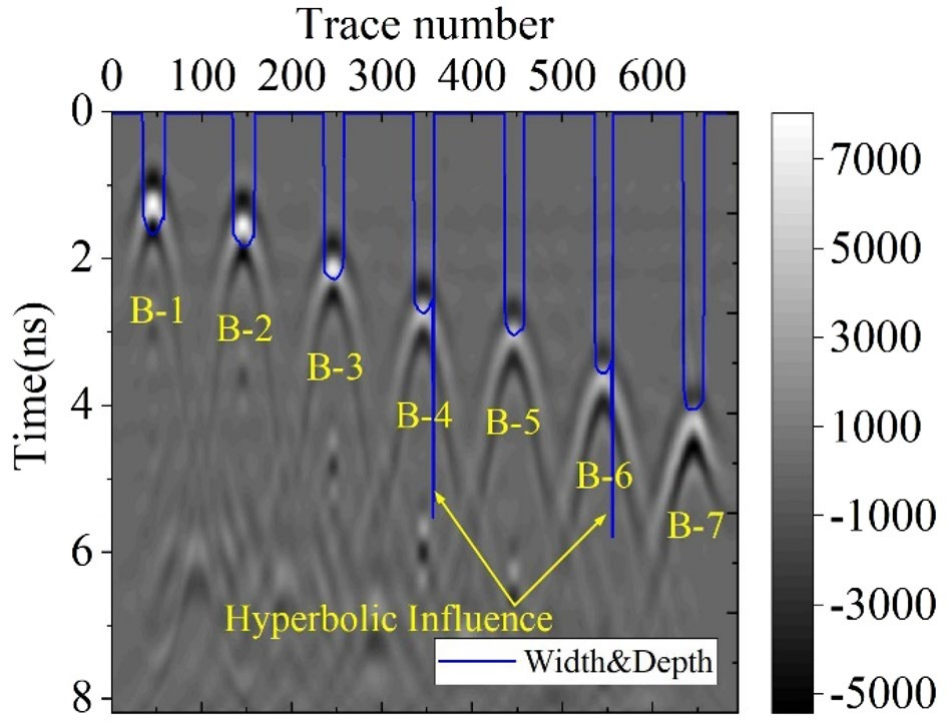
Depth and width detecting results.

After the void traces were determined, we used depth extraction algorithm to get the depth for void area. After the depth information was found, we overlapped the width and depth on the original B-scan, the result was shown in Fig. 14. The original Ricker wave is positive phase, thus the GPR wave in void area is in phase with the incident wave. In Fig. 14, almost all the void areas were correctly located at the white band area. However, there are two misjudgement area in B4 and B6 void area due to hyperbolic phenomena between adjacent void area, this interface could be filtered by adding weight function. The test result indicates that our proposed algorithm can be effectively for void area identification.

**Figure 15:**
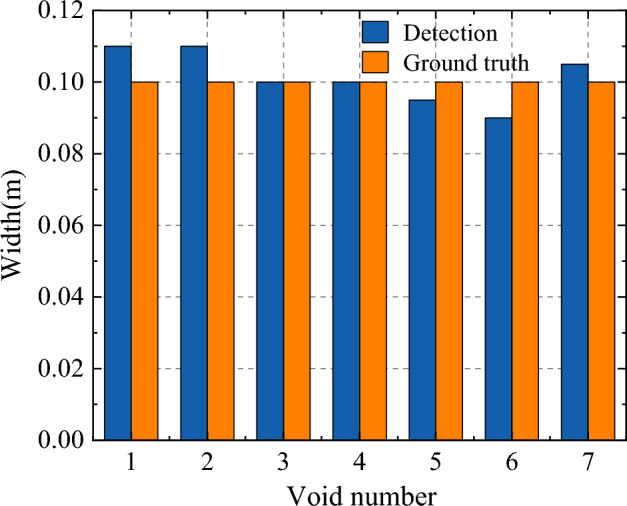
Compared result with void area detection and groundtruth.

We can not locate the depth range, we find the maximum energy point in time axis as void depth area, which is only the upper boudary of void area. Therefore, we only compare the width location accuracy. To evaluate the accuracy of our proposed method, the located void width was compared with the ground truth. Figure 15 shows width error, where the mean error is about 1.5%. Therefore, the proposed algorithm can effectively calculate the geometric parameters of void area, which could be used for further dynamics analysis in void area.

### Algorithm validation in lab model

**Figure 16:**
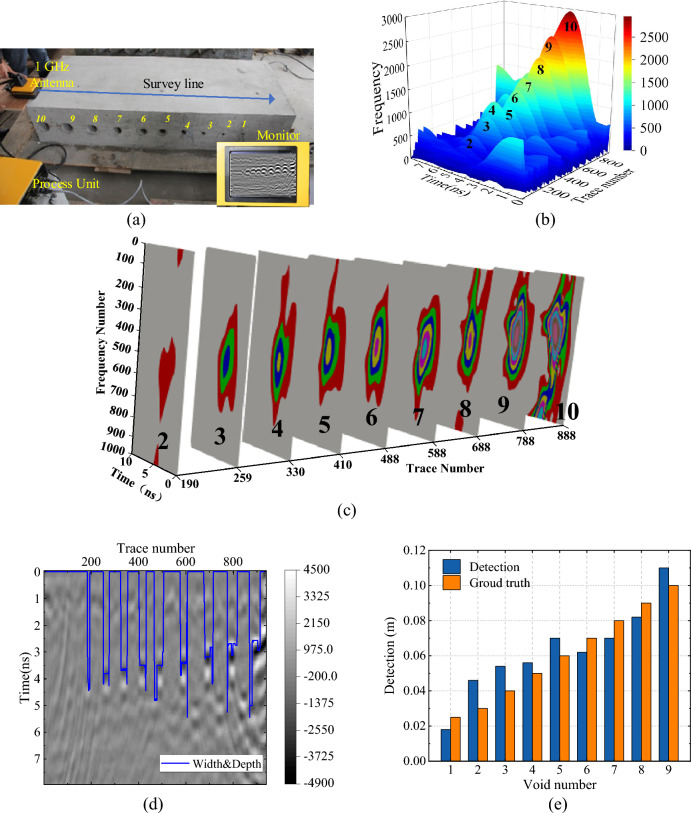
Geometric parameter extraction of void area in lab model. (**a**) The lab model. (**b**) The 3D reconstructed energy spectrum. (**c**) The CWT 3D model slice. (**d**) The depth and width detecting results. (**e**) Comparing results between detection width and ground truth.

To validate our proposed method which is also suitable for different shape, here, we didn’t stick to square void, instead, we adopted circle void. Lab model with 10 void areas shown in Fig. 16a was constructed by C30 cement with vertical dimension of 2.07 m × 0.4 m. Ten void holes with an interval of 15 cm are designed, the diameters of void areas are 100 mm, 90–30 mm, 25 mm and 16 mm in sequence, and the centre depth of holes is 20 cm from top. The USRADAR Subsurface GPR Radar Imaging System with 1 GHz antenna is used for surveying the model along the survey line as shown in Fig. 16 (a). A wood plate was used to support the antenna at the beginning to make sure hole 10 could be fully detected. This method would add noise to the GPR data, because the GPR wave is emitted and received with sphere wave, which would contain the signal of air. The sampling frequency is 16 GHz, the time window is 11.675 ns, and the sample points of each A-scan is 207. GPR data was processed by static correction, energy gain, background removal, band-pass filtering, and F-K migration method.

Figure 16b shows the 3D visualization of reconstructed energy spectrum of lab model, where 9 void areas (from hole 2 to 10) were successfully detected and viewed. Because the size of hole 1 is too small for the sample information of 1 GHz, hole 1 is not viewed in original B-scan. Figure 16c is the 3D CWT slice diagram of the lab model. The three coordinate axes are arrays corresponding to time, frequency and survey distance, respectively. The area of energy concentration is reflected through chromaticity changes, which can effectively reveal the time–frequency domain characteristics of the void area.

Similar to numerical model, we also overlap the result with width and depth information on the original B-scan and the result was shown in Fig. 16c, where the 9 holes are correctly identified. To calculate the accuracy on lab model, Fig. 16d shows the detection width with groundtruth, the error is 4.2% on the lab model, Fig. 16e shows the width error bar chart of the void disease. The error of deeper voids is greater, but as the depth decreases, the error basically shows a decreasing trend. Therefore, the proposed method can effectively extract the void width and depth information from GPR signal, which could be useful for further pavement structure safety analysis.

## Conclusions

This study proposes a method for extracting geometric parameters of void areas from GPR signals by using the CWT method. The CWT method is used to convert the GPR signal from the time domain to the time–frequency domain, where the void area exhibits energy concentration phenomena. By viewing the 3D CWT result, the void area can be clearly visulalized, and a width and depth extraction algorithm has been proposed and validated through both FDTD and lab experiments, achieving high accuracy in void width detection.

This study can be summarised as follows:

1. There are obvious differences between the time–frequency spectra of void and normal areas in the CWT results, where void signal has energy concentration phenomena while normal pavement doesn’t have.
2. The 3D CWT result provides clear visualization of the void area, which could also be used for other underground targets.
3. The proposed void width and depth extraction algorithm has been validated in both simulated and lab models, with the width detection accuracy for void areas being 1.5% and 4.2% for simulated and lab models, respectively.

In conclusion, this study presents a promising method for directly detecting the dimensions of void areas from GPR signals. However, it should be noted that this method relies on the difference in the CWT results between normal and void areas, and may not be valid if all test data comes from normal pavement without void area. Additionally, the depth information provided by this method is limited to the maximum energy depth layer of the void area due to the resolution limitations of the GPR antenna central frequency and void size. Further research is needed, including combining this method with machine learning techniques to select relevant void data, and integrating dynamics analysis of slab for determining the structural safety of void areas, which could be very useful for pavement maintenance.

### Supplementary Information

Supplementary Video 1.

## Data Availability

The data used to support the findings of this study are included within the article.
